# The immunological potency and therapeutic potential of a prototype dual vaccine against influenza and Alzheimer's disease

**DOI:** 10.1186/1479-5876-9-127

**Published:** 2011-08-01

**Authors:** Hayk Davtyan, Anahit Ghochikyan, Richard Cadagan, Dmitriy Zamarin, Irina Petrushina, Nina Movsesyan, Luis Martinez-Sobrido, Randy A Albrecht, Adolfo García-Sastre, Michael G Agadjanyan

**Affiliations:** 1 Department of Molecular Immunology, Institute for Molecular Medicine, Huntington Beach, CA 92647, USA; 2 University of California, Irvine, Institute for Memory Impairments and Neurological Disorders, Irvine, CA 92697, USA; 3 Department of Microbiology, Mount Sinai School of Medicine, New York, NY 10029 USA; 4 Department of Microbiology and Immunology, University of Rochester, Rochester, NY 14642, USA; 5 Global Health and Emerging Pathogens Institute, Mount Sinai School of Medicine, New York, NY 10029, USA; 6 Department of Medicine, Division of Infection Diseases, Mount Sinai School of Medicine, New York, NY 10029, USA

## Abstract

**Background:**

Numerous pre-clinical studies and clinical trials demonstrated that induction of antibodies to the β-amyloid peptide of 42 residues (Aβ_42_) elicits therapeutic effects in Alzheimer's disease (AD). However, an active vaccination strategy based on full length Aβ_42 _is currently hampered by elicitation of T cell pathological autoreactivity. We attempt to improve vaccine efficacy by creating a novel chimeric flu vaccine expressing the small immunodominant B cell epitope of Aβ_42_. We hypothesized that in elderly people with pre-existing memory Th cells specific to influenza this dual vaccine will simultaneously boost anti-influenza immunity and induce production of therapeutically active anti-Aβ antibodies.

**Methods:**

Plasmid-based reverse genetics system was used for the rescue of recombinant influenza virus containing immunodominant B cell epitopes of Aβ_42 _(Aβ_1-7/10_).

**Results:**

Two chimeric flu viruses expressing either 7 or 10 aa of Aβ_42 _(flu-Aβ_1-7 _or flu-Aβ_1-10_) were generated and tested in mice as conventional inactivated vaccines. We demonstrated that this dual vaccine induced therapeutically potent anti-Aβ antibodies and anti-influenza antibodies in mice.

**Conclusion:**

We suggest that this strategy might be beneficial for treatment of AD patients as well as for prevention of development of AD pathology in pre-symptomatic individuals while concurrently boosting immunity against influenza.

## Introduction

Alzheimer's disease (AD) is the most common form of dementia in the elderly which is clinically characterized by progressive loss of memory and general cognitive decline. The neuropathological features of AD include neurofibrillary tangles (NFT), deposition of soluble (monomeric, oligomeric) and insoluble fibrillar Aβ (senile plaques) forms, and neuronal loss in affected brain regions [1]. Pre-clinical and clinical trials have revealed that anti-Aβ antibodies are beneficial in clearing Aβ deposits [2-13]. The first clinical trial of active immunization against Aβ was of the vaccine AN 1792, which comprised of fibrillar Aβ_42 _formulated in a strong Th1-type biasing adjuvant, QS21. Patients treated with this vaccine were suffering mild-to-moderate AD. The trial was halted due to development of meningoencephalitis in some of the patients, which was believed to be associated with anti-Aβ specific T cell immune responses [8,9,14-16]. One possible way to avoid these side effects is the replacement of the self-T helper epitope(s) present in the Aβ_42 _peptide by a foreign epitope(s) while leaving self-B cell epitope(s) of Aβ_42 _intact. Another important, but overlooked, result from the AN-1792 clinical trial was that the majority of AD patients generated only low titers of anti-Aβ antibodies, and approximately 50% of the patients failed to produce a measurable antibody response [12,17]. The cause of the low anti-Aβ antibody titers and non-responsiveness observed in AN-1792 trial could be due to immune tolerance induced by self-Aβ_42 _antigen. The mammalian immune system normally fails to generate antibodies specific to self-molecules; however, B cell tolerance is not rigorous, while T cell tolerance is more stringent [18,19]. Previously we suggested that replacement of the Th cell epitope of Aβ_42 _by a foreign Th epitope will help to overcome not only T cell tolerance induced by self antigen, but also side effects caused by autoreactive T cells. In our previous work we generated peptide- and DNA-based epitope vaccines based on amyloid-specific B-cell epitopes Aβ_1-15 _or Aβ_1-11 _attached to the promiscuous foreign Th epitope pan HLA DR-binding peptide (PADRE) and demonstrated the feasibility of this strategy in wild-type [20-22] and APP/Tg mice [23-25]. In this study we hypothesized that for therapeutic purposes AD epitope vaccines could be delivered to patients by a conventional viral vaccine [26]. Specifically, chimeric influenza viruses expressing the B cell epitope of Aβ may not only induce anti-viral immunity, but also generate higher titers of anti-Aβ antibodies in adult individuals with pre-existing influenza virus-specific memory Th cells. Accordingly, we generated and tested for the first time the immunogenicity and protective efficacy of chimeric inactivated flu virus vaccines expressing 1-7 or 1-10 aa of Aβ_42 _(flu-Aβ_1-7 _and flu-Aβ_1-10_) in mice and demonstrated that these dual vaccines induced therapeutically potent anti-Aβ and anti-influenza antibodies.

## Materials and methods

### Mice

Female, 5-6 week-old C57Bl/6 mice were obtained from the Jackson Laboratory (MN). All animals were housed in a temperature- and light cycle-controlled animal facility at the Institute for Memory Impairments and Neurological Disorders (MIND), University of California Irvine (UCI). Animal use protocols were approved by the Institutional Animal Care and Use Committee of UCI and were in accordance with the guidelines of the National Institutes of Health.

### Generation and purification of chimeric virus

**Figure 1:**
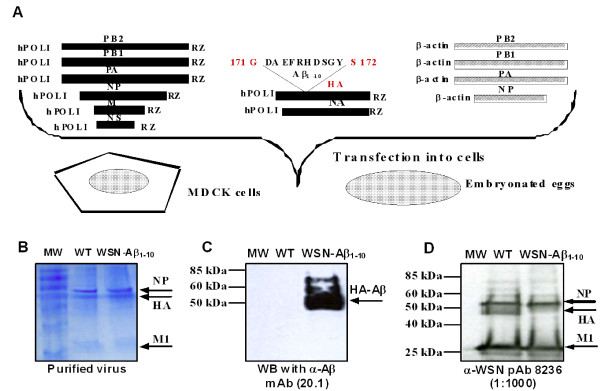
**Preparation of chimeric virus: *(A) *Schematic presentation of the rescue strategy of WSN-Aβ**_**1-10 **_**chimeric virus**. *(B) *SDS-PAGE and coomassie staining of purified chimeric (WSN-Aβ_1-10_) and wild-type (WT) viruses. *(C) *WB analysis of purified virus using anti-Aβ antibody revealed the chimeric HA-Aβ_1-10 _protein of the correct size. *(D) *Proteins corresponding to NP, HA and M1 were detected in WB analysis of purified virus using anti-WSN polyclonal serum.

Figure 1A illustrates the plasmid-based reverse genetic rescue system [26,27] used to generate chimeric influenza A/WSN/33 (H1N1) viruses expressing B cell epitopes Aβ_1-10 _(WSN-Aβ_1-10_), or Aβ_1-7 _(WSN-Aβ_1-7_) from Aβ_42_. This system includes four protein expression plasmids encoding the three influenza virus polymerase proteins (PB1, PB2 and PA) and nucleoprotein (NP), plus eight transcription plasmids encoding the eight viral gene segments. Sequences encoding B cell epitope of amyloid-β were cloned into the HA segment near the receptor binding site. Chimeric and wild-type viruses were rescued in Madin-Darby canine kidney (MDCK)/293T cell co-cultures, and the identity of the rescued viruses was confirmed by RT-PCR and restriction/sequence analysis of the HA gene segment containing the engineered foreign sequence as previously described [27]. Chimeric viruses were further grown in embryonated 10 day-old hen eggs. Viruses were purified from allantoic fluid by centrifugation through a 30% sucrose cushion. Protein concentration in purified virus samples was determined by the Bio-Rad protein assay (Bio-RAD, CA) and the purity of the samples was analyzed by SDS-PAGE (Bio-RAD, CA). The protein bands were visualized by coomassie blue staining.

### Western Blotting and Dot Blot Assay

Presence of Aβ epitope in WSN-Aβ_1-10 _or WSN-Aβ_1-7 _was confirmed by Western blot using anti-Aβ 20.1 monoclonal antibody (gift from Dr. Van-Nostrand, Stony Brook University). Influenza proteins NP, HA and M1 were visualized by staining with rabbit polyclonal anti-WSN serum (gift of Drs. Thomas Moran and Peter Palese, Mount Sinai School of Medicine). Western Blot was done as described in [28].

Binding of anti-Aβ_1-10 _sera to different forms of Aβ_42 _peptide was analyzed by Dot Blot assay. Briefly, we applied 1 μl of monomeric, oligomeric, or fibrillar forms of Aβ_42 _and irrelevant peptide (100 μM each) to a nitrocellulose membrane as described [24]. After blocking and washing, the membranes were probed with sera of mice immunized with either WSN-Aβ_1-10 _or WSN-WT formalin-inactivated virus vaccines, or with antibodies 6E10 specific for Aβ N-terminal region spanning aa 3-8 (1:3000; Covance Inc., NJ) and anti-oligomer A11 (1:500; Sigma-Aldrich, MO). Sera were used at dilution 1:200. The membranes were incubated with appropriate horseradish peroxidase-conjugated anti-mouse or anti-rabbit (only for A11) antibodies (1:1000; Santa Cruz Biotechnology, Inc., CA). Blots were developed using Luminol reagent (Santa Cruz Biotechnology, Inc., CA) and exposed to HyBlot CL Autoradiography Film (Denville Scientific Inc., NJ).

### Immunofluorescence

Expression of Aβ epitopes by chimeric viruses was analyzed by immunofluorescence of infected cells. Briefly, confluent MDCK monolayers were infected with wild-type (WSN-WT) influenza virus or chimeric viruses WSN-Aβ_1-10 _or -Aβ_1-7 _. Twelve hours post-infection cells were washed with PBS, fixed with 1% paraformaldehyde, permeabilized with 0.1% Triton X-100, blocked with 1% BSA, and then incubated with anti-Aβ (20.1) or anti-HA (2G9) MoAb. Infected cells were then incubated with a secondary anti-mouse FITC-conjugated antibody and visualized under a fluorescence microscope at ×20 magnification.

### Hemagglutination inhibition assay

Hemagglutination inhibition (HI) assays were performed using standard methods [29]. Receptor-destroying enzyme (*Vibrio cholera *filtrate; Sigma-Aldrich, MO)-treated serum as well as the anti-Aβ 20.1, anti-HA (2G9; gift of Drs. Thomas Moran and Peter Palese, Mount Sinai School of Medicine) and irrelevant anti-IRF3 antibodies (Invitrogen, CA) were used in these assays. Briefly, two fold dilutions of the indicated monoclonal antibodies or RDE-treated serum from immunized and control mice were prepared in saline solution. The diluted monoclonal antibodies or serum were then incubated with 8 hemagglutination assay (HA) units of wild-type WSN or chimeric virus. After 1 h incubation at room temperature, chicken red blood cells (RBC) were added to each well (final concentration of 0.5%) and incubated for 40 minutes on ice. The HI titer is expressed as the reciprocal of the highest dilution of serum able to inhibit hemagglutination.

### Preparation of viral stocks and immunization of mice

Viruses were grown in MDCK cells using DMEM containing 0.3% BSA, 1 μg Trypsin-TPCK/mL, penicillin, and streptomycin. After 48 h post-infection, the supernatants were collected and the viruses were pelleted by centrifugation at 25K rpm for 2 h on a 30% sucrose cushion (NTE buffer; 100 mM NaCl; 10 mM Tris-HCl, pH 7.4; 1 mM EDTA). The pellets were resuspended in NTE buffer and re-pelleted by centrifugation at 25K for 90 min in NTE buffer. The pellets were resuspended to 1 mg/ml concentration and inactivated using formaldehyde for 2 days at 4°C. To confirm complete inactivation of virus, formaldehyde treated viruses were injected into 10 d old embryonated eggs and viral replication was examined by hemagglutination assay. Mice were immunized with indicated amount of inactivated viruses formulated in Quil A adjuvant administrated subcutaneously (s.c.) at biweekly intervals. Sera were collected 12 days after each immunization.

### Detection of anti-Aβ and anti-HA antibody responses using ELISA

Concentration of anti-Aβ antibody in sera of immunized and control mice was measured as described previously [21]. Briefly, wells of 96-well plates (Immulon II; Dynax Laboratories, VA) were coated with 2.5 μM soluble Aβ_42 _(pH 9.7, o/n, and 4°C) or 10 μg/ml protein from inactivated WSN-WT virus. Wells were then washed and blocked, and sera from experimental mice were added to the wells at different dilutions. After incubation and washing, HRP-conjugated anti-mouse IgG (Jackson ImmunoResearch Laboratories, ME) was used as secondary antibody. Plates were incubated and washed, and the reaction was developed by adding *3,3',5,5'tetramethylbenzidine *(TMB) (Pierce, IL) substrate solution and stopped with 2M H_2_SO_4_. The optical density (OD) was read at 450 nm (Biotek, Synergy HT, VT), and anti-Aβ antibody concentrations were calculated using a calibration curve generated with 6E10 monoclonal antibody (Signet, MA). In order to determine half-max binding values of anti-viral antibodies we plotted the OD_450 _values against the serum dilution as described [30,31]. From this plot we determined half-maximal antibody titers (HMAT) by dividing the highest OD_450 _value in the dilution range of each serum sample by two. Initial dilution of sera in these experiments was 1:500 and they were serially diluted up to 1:500000. All anti-Aβ concentrations and HMAT were determined in individual mice.

### Detection of Aβ plaques in human brain tissues

Sera from immunized mice were screened for the ability to bind to human Aβ plaques using 50 μm brain sections of formalin-fixed cortical tissue from a severe AD case (received from Brain Bank and Tissue Repository, MIND, UC Irvine) using immunohistochemistry as described previously [20]. A digital camera (Olympus, Tokyo, Japan) was used to capture images of the plaques at an × 4 magnification. The binding of anti-Aβ sera to the β-amyloid plaques was blocked by 2.5 mM of Aβ_42 _peptide as described [20].

### Neurotoxicity Assay

Cell culture MTT assay was performed as described previously with minor modifications [24,32]. Human neuroblastoma SH-SY5Y cells (ATCC, VA) were used and aliquoted into 96-well plates (Immulon II; Dynax Laboratories, VA) at approximately 2 × 10^4 ^cells per well in 100 ml of medium (45% DMEM, 45% Ham's modification of F-12, 10% FBS and 2 mM L-glutamine) and incubated for 24 h in 5% CO_2 _atmosphere at 37°C to allow attachment to the bottom of the wells. Aβ oligomers and fibrils were prepared as we described previously [24]. Aβ_42 _oligomers and fibrils were incubated alone or with immune sera from WSN-Aβ_1-10 _(experiment) or WSN-WT (control) immunized mice for 1 h at room temperature with occasional mixing to ensure maximal interaction. After incubation, the peptide/immune sera mixtures were diluted into culture media so that the final concentration of peptide and antibodies was 2 μM and 0.2 μM, respectively. This media was then added (100 μl) to SH-SY5Y cells. The treatment time was 18 h. Untreated controls were run in parallel. Following incubation, neurotoxicity was assayed using the MTT assay according to the manufacturer's instructions (Promega Corp., WI). The absorbance at 570 nm was measured by Synergy HT Microplate reader (Biotek, VT). Cell viability was calculated by dividing the absorbance of wells containing samples by the absorbance of wells containing medium alone.

### Statistical Analysis

Statistical parameters (mean, standard deviation (SD), significant difference, etc.) were calculated using Prism 3.03 software (GraphPad Software, Inc., CA). Statistically significant differences were examined using a t-test or analysis of variance (ANOVA) and Tukey's multiple comparisons post-test (a P value of less than 0.05 was considered significant).

## Results

### Generation and characterization of chimeric viruses expressing Aβ_1-10 _or Aβ_1-7 _peptides

Previous approaches to develop AD active vaccines based on full-length β-amyloid have resulted in pathological autoimmunity [8,9,14-16]. To improve the safety profile of AD vaccines, we have constructed chimeric influenza virus A/WSN/33 (H1N1) expressing B cell epitopes of Aβ_42_, Aβ_1-10 _(WSN-Aβ_1-10_) and Aβ_1-7 _(WSN-Aβ_1-7_) using plasmid-based reverse genetic techniques described above. Influenza virus contains 200-300 molecules of HA per virion, with each of them possessing 5 antigenic sites that induce majority of neutralizing antibody responses [33]. On the other hand, the immunodominant B cell epitope of Aβ_42 _has been mapped to the N terminus of this peptide [30,34-40] and, importantly, these peptides do not possess T helper epitope/s [35,41]. Accordingly, Aβ_1-10 _(Figure 1A) and Aβ_1-7 _(data not shown) epitopes of Aβ_42_, were inserted into one of five HA antigenic sites between amino acids 171 and 172. The other four antigenic sites of HA remained unaltered so they could induce virus-neutralizing antibodies. Generated chimeric viruses were purified and the expression of inserted antigens was tested. As shown in Figure 1B, coomassie staining of SDS-PAGE resolved purified viruses revealed that the purity of both chimeric (WSN-Aβ_1-10_) and wild-type (WSN-WT) viruses reached to > 90%. Immunoblot analysis conducted with anti-Aβ monoclonal antibody (20.1) demonstrated that chimeric, but not WT, virus expressed an Aβ peptide incorporated into the viral protein (HA) (Figure 1C), while both viruses expressed HA, NP and M1 proteins detected with anti-WSN antibodies (Figure 1D). Of note, to make it simple, only data with WSN-Aβ_1-10_, but not WSN-Aβ_1-7 _were presented in Figure 1.

**Figure 2:**
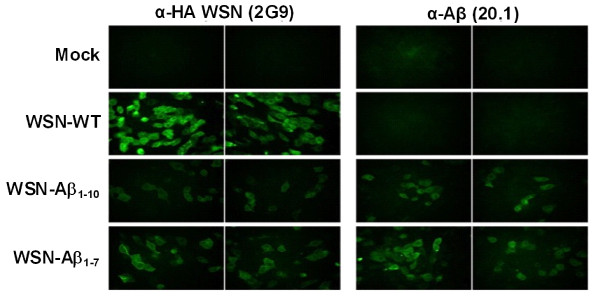
**Expression of β-amyloid B cell epitopes by chimeric influenza virus WSN (WSN-Aβ**_**1-10 **_**and WSN-Aβ**_**1-7**_). MDCK cells infected with WSN-Aβ_1-10 _and WSN-Aβ_1-7 _were positive for immunostaining with anti-Aβ and anti-HA antibodies, whereas cells infected with WSN-WT were positive only with anti-HA antibody.

**Figure 3:**
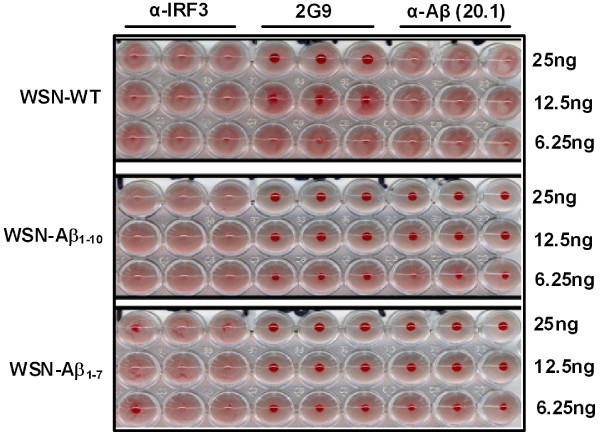
**Anti-HA antibodies inhibited agglutination of RBC by both wild-type and chimeric influenza viruses, while anti-Aβ antibodies only inhibited agglutination of RBC by the chimeric virus**.

Next, we compared the ability of WT virus and Aβ peptide expressing chimeric viruses to infect the host cells *in vitro *by immunofluorescence assay. MDCK cells mock-infected or infected with WSN-WT, WSN-Aβ_1-10 _or WSN-Aβ_1-7 _were stained with either anti-Aβ (20.1) or anti-HA (2G9) monoclonal antibodies (Figure 2.). Importantly, WSN-WT-infected cells stained positive only with anti-HA antibody. WSN-Aβ_1-10 _or WSN-Aβ_1-7 _infected cells stained positive for Aβ and anti-HA (Figure 2). These data supported biochemical results presented in Figure 1 and also suggested that the insertion of Aβ peptide into the HA molecule did not perturb the infectivity of the chimeric flu virus. A hemagglutination inhibition (HI) assay (Figure 3) was next conducted to analyze the impact of the Aβ insertion in recognition of the HA by neutralizing antibodies. Interestingly, anti-Aβ monoclonal antibody (20.1) inhibited hemagglutination of chicken red blood cells (RBC) by WSN-Aβ_1-10 _or WSN-Aβ_1-7 _viruses, but not by WSN-WT (Figure 3). The anti-HA monoclonal antibody (2G9) inhibited hemagglutination of RBC by chimeric and wildtype viruses, whereas a negative control antibody specific for IRF3 did not inhibit hemagglutination. These data demonstrate that (i) the Aβ epitope is displayed on the virus surface allowing for the recognition by anti-Aβ antibodies and (ii) the insertion of Aβ peptide did not drastically change the conformation of the HA molecule and did not disturb its functional ability.

### WSN-Aβ_**1-10 **_is more immunogenic than WSN-Aβ_1-7_

To evaluate the ability of chimeric influenza viruses expressing Aβ_1-10 _and Aβ_1-7 _peptides to induce anti-Aβ antibody responses, C57Bl/6 mice were immunized with 20 μg/mouse purified inactivated chimeric viruses (formulated in a strong Th1 type adjuvant, QuilA, three times with two weeks interval (Table 1*, Study 1*).

**Table 1:** Design of immunization studies in wild-type mice

Study	Group	Immunogen	Dosage (μg/mouse)	Total number of Immunizations
***Study 1***	1	WSN-WT	20	3
	
	2	WSN-Aβ_1-7_	20	3
	
	3	WSN-Aβ_1-10_	20	3

***Study 2***	1	WSN-WT	5	3
	
	2	WSN-WT	25	3
	
	3	WSN-WT	50	3
	
	4	WSN-Aβ_1-10_	5	3
	
	5	WSN-Aβ_1-10_	25	3
	
	6	WSN-Aβ_1-10_	50	3

***Study 3***	1	WSN-WT	50	6
	
	2	WSN-Aβ_1-10_	50	6

**Figure 4:**
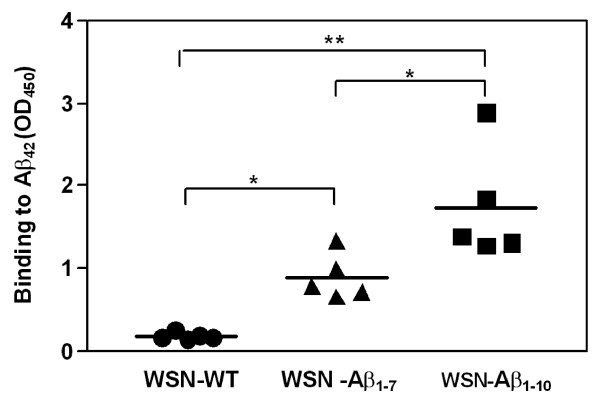
**Mice immunized with killed WSN-Aβ**_**1-10 **_**virus generated significantly higher anti-Aβ**_**42 **_**specific antibodies compared with that in mice immunized with WSN-Aβ**_**1-7**_. Anti-Aβ antibody responses were measured in sera of individual mice immunized 3 times with indicated viruses at dilution 1:200. Lines represent the average (n = 5, *P < 0.05; **P < 0.01).

Control groups of mice were immunized with 20 μg/mouse of inactivated purified WSN-WT. An Aβ-specific ELISA revealed that both chimeric influenza viruses expressing Aβ_1-10 _or Aβ_1-7 _induced anti-Aβ antibody responses after three immunizations; however, antibody responses were significantly stronger for WSN-Aβ_1-10 _immunized mice as compared to WSN-Aβ_1-7 _immunized mice (Figure 4). No anti-Aβ response was seen in the control group of mice immunized with WSN-WT (Figure 4). Based on the higher ELISA titer, the chimeric influenza virus WSN-Aβ_1-10 _was chosen for further experiments.

### Humoral immune responses generated by WSN-WT and WSN-Aβ_1-10 _vaccines are dose-dependent

**Figure 5:**
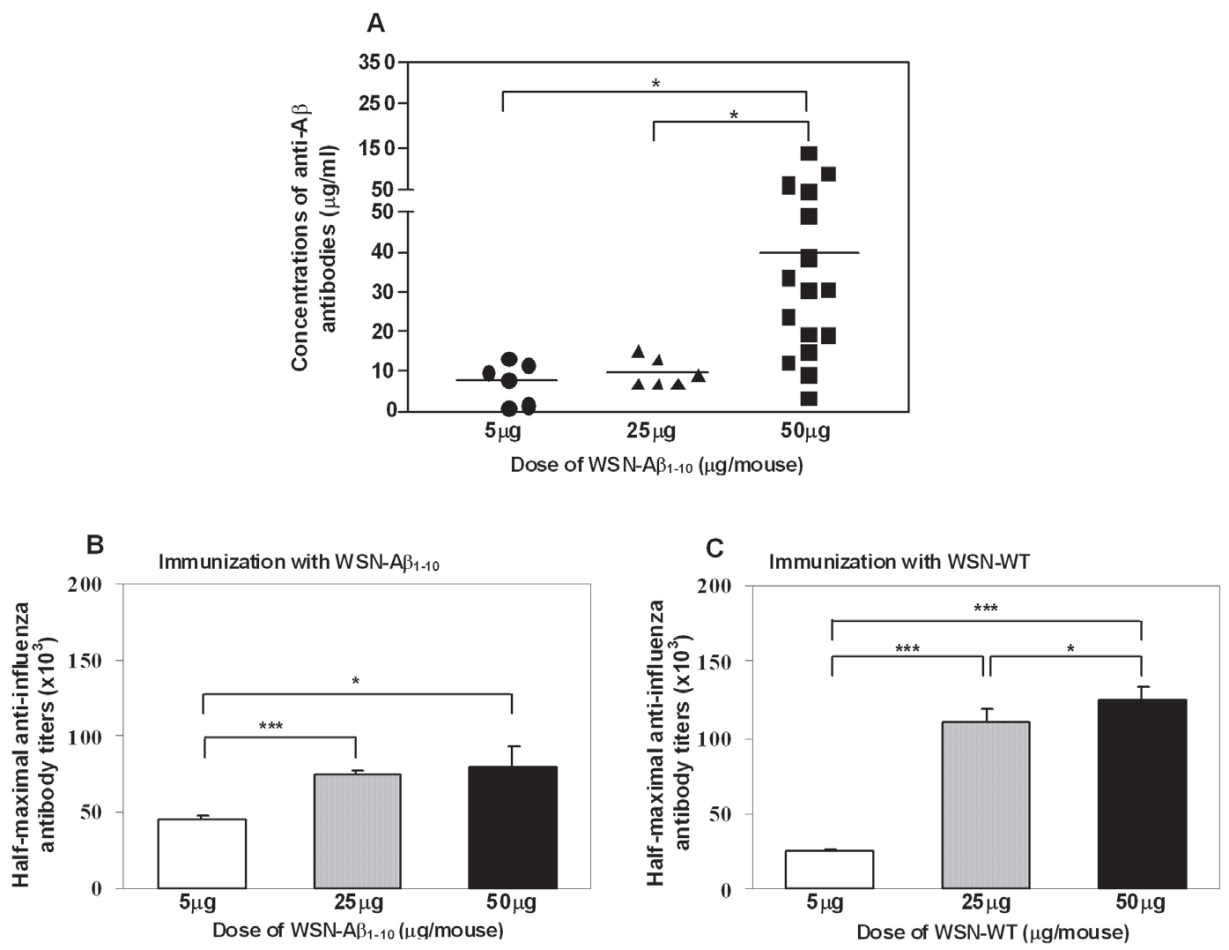
**Anti-Aβ and anti-WSN immune responses in mice immunized with different doses of WSN-Aβ**_**1-10 **_**and WSN-WT:** Anti-Aβ *(A) *and anti-WSN *(B, C) *antibodies were analyzed in sera of individual mice immunized 3 times with indicated doses of killed WSN-Aβ_1-10 _and WSN-WT viruses formulated in Quil A. Lines and error bars indicate the average ± s.d. (n = 6 for groups immunized with 5 and 25 μg and n = 16 for groups immunized with 50 μg killed viruses (*P < 0.05; ***P < 0.001).

Next we investigated the effects of an increased antigen dose on generation of anti-Aβ and anti-influenza antibodies (Table 1, *Study 2*). C57Bl/6 mice were immunized with three different doses (5 μg, 25 μg and 50 μg per mouse) of WSN-Aβ_1-10 _or WSN-WT. Humoral immune responses were evaluated in all groups after the third immunization (Figure 5). Immunizations with 5 μg/mouse or 25 μg/mouse doses of WSN-Aβ_1-10 _induced relatively low levels of anti-Aβ antibodies (7.47 ± 5.29 μg/ml and 9.47 ± 3.52 μg/ml, respectively). However, 50 μg/mouse dose of WSN-Aβ_1-10 _(40.01 ± 35.66 μg/ml) induced strong anti-Aβ antibody response that was significantly higher (P ≤ 0.05) than that in mice vaccinated with 5 μg/mouse or 25 μg/mouse doses (Figure 5A). Both 25 μg/mouse and 50 μg/mouse doses of WSN-Aβ_1-10 _induced significantly higher (P ≤ 0.05) titers of anti-WSN antibody (~75,000 and ~80,000, respectively) than that in mice immunized with 5 μg/mouse dose of WSN-Aβ_1-10 _(~45,000) (Figure 5B). Of note, although the anti-WSN antibody response was slightly higher in mice immunized with 50 μg WSN-Aβ_1-10 _compared with that in mice immunized with 25 μg WSN-Aβ_1-10_, this difference was not significant. In case of immunization with WSN-WT virus the dose-dependent nature of humoral response was more evident. 50 μg/mouse of WSN-WT induced significantly higher titers of anti-influenza antibodies (~125,000) than 25 μg/mouse (~110,000, P ≤ 0.05) and 5 μg/mouse doses (~25,000, P ≤ 0.001), respectively (Figure 5C). Thus, mice immunized with 50 μg of inactivated chimeric virus generated the strongest anti-amyloid and anti-influenza humoral immune responses and this dose of vaccine have been used in our further experiments described below.

### Kinetics of antibody responses in mice immunized with WSN-WT and WSN-Aβ_1-10 _viruses

**Figure 6:**
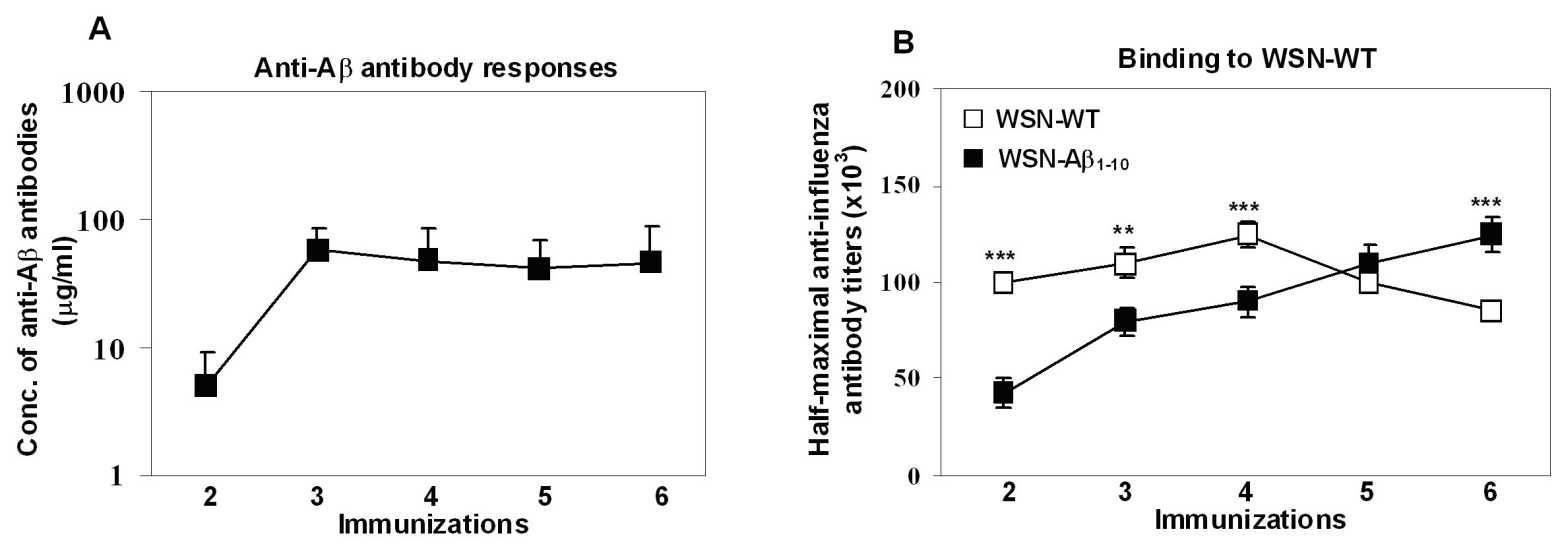
**Kinetics of anti-Aβ *(A) *and anti-WSN-WT antibody responses *(B) *in mice immunized with 50 μg/mouse of WSN-Aβ**_**1-10 **_**and WSN-WT viruses**. Concentration of anti-Aβ antibodies and half-maximal titers (HMAT) of anti-WSN-WT antibodies were analyzed in individual mice. HMAT was determined in the sera of individual mice by dividing the highest OD_450 _value in the dilution range of each sample by two. Initial dilution of sera in these experiments was 1:500 and they were serially diluted up to 1:500000. Error bars indicate the average ± s.d. n = 16 and n = 8 in groups immunized with WSN-Aβ_1-10 _and WSN-WT viruses respectively (**P < 0.01, ***P < 0.001).

The kinetics of anti-Aβ antibody and anti-influenza antibody responses in mice vaccinated with WSN-Aβ_1-10 _or WSN-WT were analyzed to determine the minimal number of vaccinations required to achieve maximal humoral responses and to determine if a correlation existed between the kinetics of Aβ antibody and influenza virus HA responses. Two groups of mice were immunized six times biweekly with inactivated WSN-Aβ_1-10 _or WSN-WT formulated in Quil A adjuvant (Table 1, *Study 3*). The concentration of anti-Aβ antibodies was measured in sera of mice after each immunization starting from the second immunization (Figure 6A). The highest Aβ antibody titer was detected after the 3^rd ^immunization with WSN-Aβ_1-10 _(56.47 ± 30.18 μg/ml). Further immunizations did not change the level of anti-Aβ antibodies as the titers reached a plateau (after 6^th ^immunization titers were still the same = 46.43 ± 42.66 μg/ml). As expected, WSN-WT immunized mice did not show any detectable anti-Aβ antibody responses (data not shown).

Importantly, immunization with WSN-Aβ_1-10 _elicited also high titers of anti-WSN antibodies after the second immunization, and these titers became even higher after each subsequent immunization reaching up to ~125,000 after six immunizations (Figure 6B). In contrast, WSN-WT immunization elicited the highest level of anti-influenza antibody much quicker (after 4^th ^immunization titer of antibodies was ~125,000), which then decreased after 5^th ^and 6^th ^immunizations (Figure 6B). Thus, although after early immunizations the titers of anti-influenza antibodies were significantly higher in mice immunized with WSN-WT than with WSN-Aβ_1-10_, the pattern was changed after further immunizations. Interestingly, after the 6th immunizations titers of anti-influenza antibody elicited by WSN-Aβ_1-10 _were significantly higher than that elicited by WSN-WT.

### Anti-Aβ and anti-influenza antibodies are therapeutically potent

**Figure 7:**
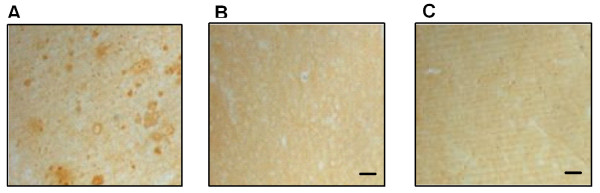
**Therapeutic potency of anti-Aβ antibody generated in mice immunized with WSN-Aβ**_**1-10**_: *(A) *Immune sera generated after immunization with killed WSN-Aβ_1-10 _(at dilution 1:600) bound to the brain sections of cortical tissues from an AD case and *(B) *this binding was blocked by pre-absorption of sera with Aβ_42 _peptide. *(C) *Immune sera generated after immunization with killed WSN-WT (at dilution 1:600) did not bind to the brain sections of cortical tissues from an AD case. Original magnification was ×4 and scale bar was 200 μm.

To show the therapeutic potential of dual chimeric vaccine we first analyzed binding of antisera to Aβ plaques in brain tissue from an AD case. As we expected from our previous studies [20,22,24], sera generated after immunizations of mice with WSN-Aβ_1-10 _bound to β-amyloid plaques very well (Figure 7A). This binding was specific to Aβ since it was blocked by pre-absorption of antisera with Aβ_42 _peptide (Figure 7B). As one could expect from data presented above, sera obtained from mice immunized with WSN-WT did not bind to Aβ deposits in AD brain tissue at all (Figure 7C).

**Figure 8:**
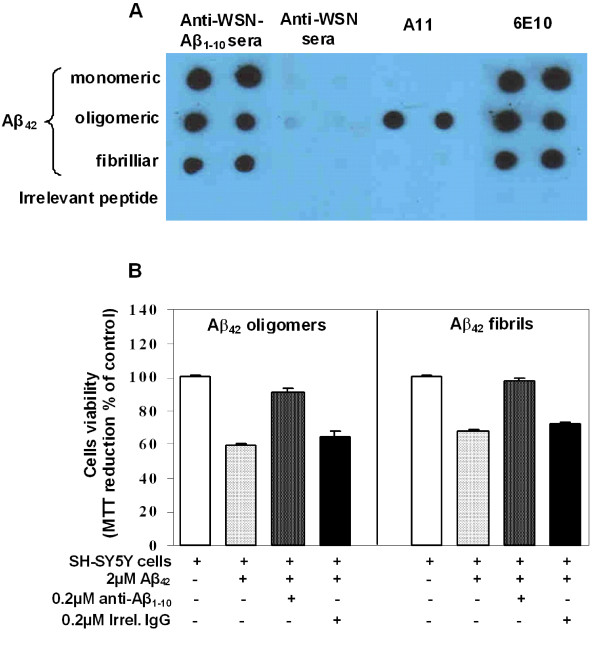
**Antibodies generated in mice immunized with dual vaccine, WSN-Aβ**_**1-10 **_**bind to Aβ**_**42 **_**and inhibit its neurotoxicity:** (A) Sera isolated from WSN-Aβ_1-10_, but not WSN-WT vaccinated mice at dilution 1:200 bound to all species of Aβ_42 _peptide, including oligomers recognized by A11 oligomer-specific antibodies. Control monoclonal 6E10 antibody bound to all forms of Aβ_42 _peptide. (B) Anti-Aβ_1-10 _inhibits Aβ_42 _fibrils- and oligomer-mediated toxicity. Human neuroblastoma SH-SY5Y cells were incubated with Aβ_42 _oligomers and Aβ_42 _fibrils, in the presence or absence of anti-Aβ_1-10 _antibody or irrelevant mouse IgG. Control cells were treated with the vehicle, and cell viability was assayed in all cultures using the 3-(4,5-dimethylthiazol-2-yl)-2,5-diphenyltetrazolium bromide assay. Data were collected in four replicate and was expressed as a percentage of control ± s.d.

The important feature of functional anti-Aβ antibody is the binding to all species of Aβ_42 _peptide and inhibition of cytotoxic effect of Aβ_42 _oligomers and fibrils on human neuroblastoma SH-SY5Y cells. We demonstrated that immune sera from mice immunized with WSN-Aβ_1-10 _bound very well to monomeric, oligomeric and fibrillar forms of Aβ_42 _peptide in a dot blot assay (Figure 8A). Thus, we confirmed that WSN-Aβ_1-10 _vaccine induced anti-Aβ antibodies capable of binding not only to Aβ_42 _oligomers and fibrils *in vitro*, but also to plaques of AD case. These data suggested that anti-Aβ antibody generated by WSN-Aβ_1-10 _vaccine is therapeutically potent and might exhibit a protective effect on Aβ-induced neurotoxicity. To test that, we performed *in vitro *assessment using human neuroblastoma SH-SY5Y cells. The data showed that both Aβ_42 _fibrils and oligomers are cytotoxic, reducing cell viability to about 67.7% and 59.8%, respectively (Figure 8B). Pre-incubation of Aβ_42 _fibrils with immune sera from WSN-Aβ_1-10 _vaccinated mice resulted in the rescue of cell viability to maximum level (~97.5%). Similarly, pre-incubation of Aβ_42 _oligomers with anti-Aβ_1-10 _antibody increased cell viability to approximately 90.9%. In contrast, pre-incubation of both Aβ_42 _species with immune sera from WSN-WT immunized mice (control) did not rescue cells from oligomer or fiber-mediated cell death. These data suggest that anti-Aβ_1-10 _antibody generated by WSN-Aβ_1-10 _chimeric vaccine inhibits Aβ_42 _fiber-mediated neurotoxicity and alleviates oligomer-mediated toxicity *in vitro*.

**Figure 9:**
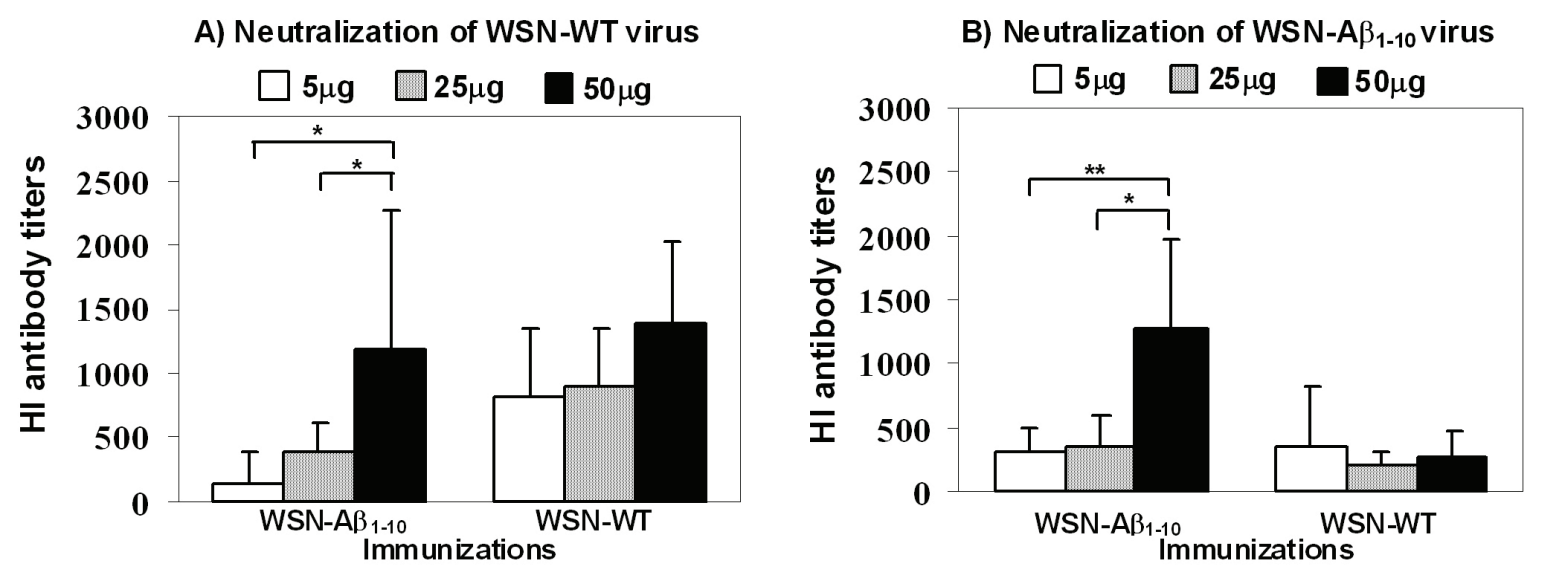
**Antibodies generated in mice immunized with dual vaccine, WSN-Aβ**_**1-10 **_**neutralize both WSN-WT (*A*) and WSN-Aβ**_**1-10 **_**(*B*) viruses**. Titers of HI antibody against WSN-WT (*A*) or WSN-Aβ_1-10 _(*B*) viruses were measured in individual mice (n = 6/per group) after 3 immunizations. The statistical difference between each group was determined (*P < 0.05; **P < 0.01).

**Figure 10:**
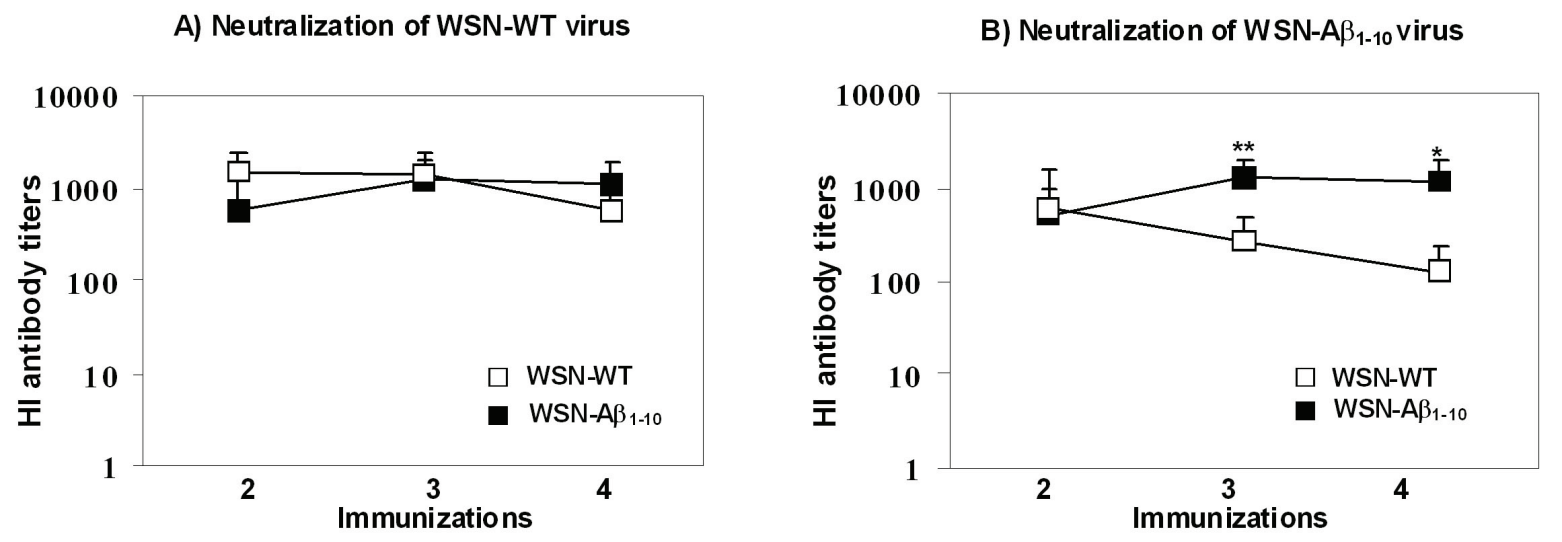
**Virus neutralization titers of sera generated after 2, 3 and 4**^**th **^**immunizations with dual vaccine and WSN-WT are the same**. HI titers against WSN-WT *(A) *and WSN-Aβ_1-10 _*(B) *were evaluated in sera of individual mice immunized after 2, 3, and 4 immunizations with WSN-WT (close sq) or WSN-Aβ_1-10 _(open sq). Error bars indicate the average ± s.d. for mice immunized with WSN-Aβ_1-10 _(n = 16) or WSN-WT (n = 8) (*P <0.01; **P < 0.01).

Next in order to understand the dual potency of WSN-Aβ_1-10 _it was important to analyze the anti-viral efficacy of antibodies generated by the chimeric vaccine. The level of neutralizing anti-viral antibodies in immunized mice was measured using the HI assay described above. HI antibody titers were determined in groups immunized with different doses (5 μg, 25 μg, or 50 μg) of chimeric and wildtype viruses against both types of viruses: WSN-Aβ_1-10 _and WSN-WT (Table 1, *Study 2*). After 3 immunizations all mice had measurable titers (> 1:40) of HI antibodies against both viruses. The titers of HI antibody in pre-bleed sera were < 1:10 (data not shown). Immunization with 50 μg/mouse WSN-Aβ_1-10 _induced significantly higher titers of HI antibodies against both wild-type and chimeric viruses than the immunizations by 5 μg/mouse and 25 μg/mouse doses of WSN-Aβ_1-10 _(P ≤ 0.05 and P ≤ 0.01, respectively, Figure 9A, B). No significant differences in titers of HI antibodies against both chimeric and wild type WSN viruses were observed in mice immunized with three different doses of WSN-WT (Figure 9A and 9B). The kinetics of anti-HA neutralizing antibodies were also analyzed in the sera of mice immunized with 50 μg/mouse dosage of WSN-Aβ_1-10 _and WSN-WT (Table 1, *Study 3*). The titers of HI antibodies were measured after two, three and four immunizations against WSN-WT (Figure 10A) and WSN-Aβ_1-10 _(Figure 10B) viruses using HI assay. Both viruses elicited equal titers of functional anti-HA antibodies inhibiting hemagglutination by wild-type virus. However, titers of functional antibodies inhibiting hemagglutination by WSN-Aβ_1-10 _virus was significantly higher in mice immunized with WSN-Aβ_1-10 _than in mice immunized with WSN-WT (P ≤ 0.01 and P ≤ 0.05 after 3^rd ^and 4^th ^immunizations, respectively, Figure 10B). Thus, chimeric WSN-Aβ_1-10 _vaccine was at least as good as WSN-WT in generation of virus neutralizing antibodies, however it had an additional benefit as it also induced therapeutically potent anti-AD antibodies.

## Discussion

Different approaches that aimed to prevent Aβ over-production or accelerate its degradation are currently being developed for treatment of AD. However all available treatments have only relatively small symptomatic benefits and could not delay or halt the progression of the disease. As a result, there is no cure from AD today. A potentially powerful strategy is immunotherapy with anti-Aβ antibody that can facilitate the reduction of pathological forms of Aβ in the brain [42-52] via several pathways, including catalytic dissolution of amyloid deposits by antibodies; Fc mediated macrophage phagocytosis of amyloid; non-Fc mediate macrophage amyloid clearance; a peripheral sink, whereby Aβ is drawn out of the brain into the peripheral circulation [53,54].

The results of the first AD clinical trial using the AN-1792 vaccine confirmed that anti-Aβ antibodies are beneficial for AD patients and may at least slow the progression of a disease. However this trial raised concerns about the safety and the efficacy of the active immunization strategy with Aβ_42 _self-peptide. Although the results from the Phase I trial showed good tolerability, in the phase IIa portion of the AN-1792 immunotherapy a subset of individuals developed adverse events in the central nervous system [8-11,14-17]. Further examinations demonstrated that these adverse effects were presumably due to the infiltration of autoreactive T cells, rather than anti-Aβ antibody. In addition, the relatively low antibody titers generated even after multiple immunizations and non-responsiveness in ~80% of patients indicating that the Aβ self-antigen vaccine was not a strong immunogen, suggest that alternative immunotherapeutic strategies should be pursued.

Based on data that the immunodominant B cell epitope of Aβ_42 _has been mapped to the N-terminus of this peptide (aa spanning residues 1-5, 1-7, 1-8, 1-11, 1-15, 1-16, or 4-10) [34,35,37,39,55] and that this Aβ_1-11 _peptide does not contain a T cell epitope in mice [35] or in humans [56], we proposed to use a prototype epitope vaccine that contains the small immunodominant self-B cell epitope of Aβ in tandem with promiscuous foreign T helper cell epitope/s, in order to reduce the risk of an adverse T cell-mediated immune response to Aβ-immunotherapy [20]. The efficacy and immunogenicity of our peptide and DNA-based epitope vaccines have been previously tested in the pre-clinical trials [23-25]. Other groups of scientists and different pharmaceutical companies are working on development of epitope-based AD vaccines composed of self-Aβ B cell epitope attached to the carrier protein rather than small foreign Th epitope [57]. Another category of epitope vaccines are those based on viral-like particles (VLP) [58-61]. Incorporation of the Aβ B cell epitope into a viral capsid protein or scaffold proteins allows the expression of this epitope on the surface of VLP in a repetitive and ordered array. Such organization of the epitope may induce T cell-independent B cell activation and production of anti-Aβ antibodies of IgM isotype. On the other hand, T cell epitopes from the viral proteins may help B cells to induce T cell-dependent humoral responses and produce antibodies of other isotypes. In fact, high titers of persisting long-term anti-Aβ antibodies were induced by recombinant protein based on pyruvate dehydrogenase complex of *B. stearothermophilus *fused with Aβ_1-11 _B cell epitope. This protein self assembles *in vitro *into a high molecular mass scaffold with icosahedral symmetry exposing Aβ B cell epitope on a surface [62]. Therapeutically potent anti-Aβ antibodies (up to 1:10000 titer) were generated in APP/Tg mice using VLP based on papillomavirus [58,61], retrovirus [59], Qβ bacteriophage [58,60]. Qβ-based vaccine comprising the Aβ_1-6 _epitope (CAD106) covalently linked to VLPs [63] is currently in Phase II clinical trials conducted by Novartis. Report from Phase I trial on safety, tolerability and Aβ-specific antibody responses in a group of patients with mild to moderate AD following three subcutaneous injections of 50 μg (cohort I) and 150 μg (cohort II) CAD106 was encouraging and showed that adverse events were predominantly mild. Although CAD106 induced low titers of specific antibody with a 2-fold increase in cohorts II vs I, 16/24 and 18/22 of subjects in cohort I and cohort II, respectively, responded to the vaccine [64,65].

Our chimeric vaccine strategy described in this paper is different from VLP-based vaccines. First of all it is based on whole chimeric virus instead of non-replicative particles and therefore it could be used as either killed or live attenuated virus based vaccine. The use of chimeric influenza viruses whose backbone is widely used as a human influenza vaccine has the advantages of having quite well known antigenic properties in humans, of its immunogenicity being helped in humans by memory T cell responses against the backbone virus. More importantly, our strategy aimed to generate dual vaccine and test the feasibility of this approach.

Accordingly, we decided to take advantage from our previously developed plasmid-based reverse genetic technique [26] and generate a dual vaccine expressing the short B-cell epitope of amyloid within the HA of influenza virus. The HA and NA glycoproteins of influenza A viruses contain the major antigenic determinants of the virus responsible for the induction of neutralizing (protective) immune response. The appropriate mutations or insertions that may attenuate virus without compromising the immunogenicity of the vaccine allowed generating chimeric viruses (vectors) that can express heterologous polypeptides [66]. Because influenza viruses are potent inducers of antigen-specific B and T cell immune responses [66] they can also be attractive candidates as delivery vectors for amyloid-β B-cell epitope. In fact, previously it was shown that appropriate chimeric influenza viruses delivered heterologous small antigen (usually about 10-12 aa) into the host [67] and induced potent antibody [68] or cellular [69] immune responses specific to grafted peptide.

Here we generated and studied dual vaccines based on chimeric viruses, expressing Aβ_1-10 _or Aβ_1-7 _epitopes of Aβ_42. _These B-cell epitopes of amyloid-β were inserted between amino acids 171 and 172 of HA, while the other four antigenic sites of HA remained intact (Figure 1A). The WB analysis demonstrated that chimeric, but not WNT-WT virus expressed HA of correct size containing Aβ_1-10 _(Figure 1C) or Aβ_1-7 _(data not shown) peptides. Importantly, the insertion of Aβ into HA did not change the capability of virus to infect host MDCK cells (Figure 2) or the conformation of the HA molecule (Figure 2 and 3).

Next we decided to analyze the immunogenic potency of the chimeric virus and compare it with that of wild-type influenza virus. Purified WSN-Aβ_1-10_, WSN-Aβ_1-7_, or WSN-WT viruses (Figure 1B and data not shown) has been used for preparation of inactivated vaccines that have been formulated into Th1 type adjuvant prior to immunization of experimental and control mice. We demonstrated that WSN-Aβ_1-10 _was more immunogenic than WSN-Aβ_1-7 _(Figure 4) and it induced the highest titers of anti-amyloid and anti-viral antibodies at 50 μg/mouse dose (Figure 5). WSN-Aβ_1-10 _induced as good anti-viral humoral immune responses as WSN-WT after 3-4 immunizations (Figure 5, 6). These results support our hypothesis that chimeric influenza virus could be an excellent delivery platform for Aβ epitope, and at the same time provide T helper cell help to Aβ specific B cells. Of note, using peptide, recombinant protein and DNA based epitope vaccines we showed that Aβ_1-11 _region did not possess epitopes for H2-b and H-2d mice [20,23,25]. More importantly, it was shown that Th epitope of Aβ_42 _mapped to C-terminal region of this peptide [56]. Based on these data currently several companies are conducting Phase I/IIa studies with carriers fused with N-terminal regions of amyloid [70,71].

The data represented above implied that a dual vaccine strategy is feasible since vaccinations of mice induced strong anti-viral and anti-amyloid humoral immune responses. At the same time these results did not demonstrate the therapeutic potency of anti-influenza and anti-Aβ antibodies. To test that, we performed *in vitro *assessment using HI [29] and neurotoxicity [24,32] assays routinely used in our laboratories. These analyses showed that chimeric virus maintained the ability to induce the production of (i) virus neutralizing antibodies that inhibited the hemagglutination of red cells by the both chimeric and wild-type viruses (Figure 9, 10); and (ii) anti-Aβ antibodies that are binding to various Aβ_42 _forms (Figure 8A) and inhibiting Aβ_42 _fibrils- and oligomer-mediated toxicity of human neuroblastoma SH-SY5Y cells (Figure 8B). Data presented above suggest that anti-viral antibody could block viral infection while anti-Aβ antibody could be an effective modulator of Aβ_42 _aggregate formation regardless of the nature of the aggregated species. Indeed, anti-Aβ antibody bind not only Aβ_42 _fibrils and oligomers *in vitro*, but also Aβ plaques present in brain sections of cortical AD tissue (Figure 7).

To our knowledge this is the first attempt for generation dual vaccine based on conventional seasonal Flu vaccine and therefore designed to protect the elderly from both AD and seasonal Flu infection. Annual administration of seasonal Flu vaccine is currently proposed, therefore it is important to study the persistence of anti-Aβ antibodies and optimized schedule for vaccination with dual vaccine. However, in mice that are leaving in average 2.2-3.2 years it is not accurate testing annual vaccination strategy used for vaccination of elderly people. Thus, we are currently planning to study the doses, type of vaccine (killed or live attenuated), as well as schedule for vaccination in non-human primates, including aged animals with immunosenescence. The major complication connected with vaccination of elderly people is the poor response to the vaccines due to the immunosenescence. One possible strategy to counteract the immunosenescence is to recruit previously generated memory T cells produced during prior vaccinations and/or exposure to human pathogens. The majority of people already possess memory T cells specific for influenza due to yearly vaccinations and/or infection by virus. Thus, immunization of elderly people with our dual vaccine may in theory recruit memory T helper cells specific to influenza epitopes and induce rapid and potent anti-Aβ antibody production, while continuing to boost anti-viral cellular and humoral responses. This hypothesis is the subject of studies in progress in our laboratories.

Another important aspect of a dual vaccine is related to the safety issues. Since the majority of people including children and elderly are vaccinated with influenza vaccine yearly and the safety of this vaccine is observed for a long period of time, the chance that the dual vaccine is safe is very high. Finally, we think that the availability of a safe dual vaccine will allow the treatment of pre-symptomatic people rather than AD patients. Based on both preclinical studies and the results from the AN1792 clinical trials [70,71] we may assume that early intervention in the disease process, pre-symptomatic if possible, is likely to be significantly more beneficial than attempting to intervene in the disease process after clinical diagnosis of the disease. In addition, early intervention is likely to significantly reduce the probability of adverse events in response to active immunization [14]. We believe that the recent breakthroughs in the development of biomarkers for AD provide a hope that patients can be accurately identified while they are still in the preclinical stages of AD [72-77], which should facilitate the usage of dual vaccines before extensive neuronal damage and cerebral amyloid angiopathy has occurred in the brain in the general population. At the same time it should be mentioned that many groups including us have not observed infiltration of autoreactive T cells (presumed Th1 response that likely occurred in AN1792 vaccinated patients) in the brains after immunizations of APP/Tg or wild-type mice with the original Schenk et al. protocol [2] or with other Aβ vaccines (unless pertussis toxin widely used to induce brain T cell penetration in experimental autoimmune encephalomyelitis have been co-administered [78]). Thus, obviously only clinical trials may help us to conclude that any epitope vaccine including our chimeric flu vaccine is safe and do not induce harmful proinflammatory T cell responses in vaccinated AD patients.

## Declaration of competing interests

Authors declare that they have no competing interests. Dr. García-Sastre is named inventor of a patent filed through Mount Sinai School of Medicine that is related to the generation of recombinant influenza A viruses from plasmid DNA.

## Authors' contributions

HD contributed substantially in design of study, performed the immunization of mice, carried out immunoassays (ELISA, Dot Blot, Neurotoxicity assay). He participated in analyses and interpretation of data. He drafted the manuscript. AG has been involved in analyses and interpretation of data and statistical analysis. She helped to draft the manuscript. RC participated in preparation of chimeric viruses, purification of viral proteins and performing of hemagglutination inhibition assays. DZ cloned, generated, and characterized chimeric viruses. IP analyzed binding of antisera to Aβ plaques in brain tissue from an AD case. NM participated in immunization of mice and analyzed antibody responses using ELISA. LMS generated and characterized chimeric viruses, performed hemagglutination inhibition assays and participated in purification of chimeric viruses. RAA participated in analyses and interpretation of data. AGS helped to troubleshoot difficulties connected with experiments, helped to draft the manuscript, revised it critically for important intellectual content. MGA conceived the study, mentored primary authors, helped to analyze the data and make conclusions, prepared final version of manuscript. All authors read and approved the final manuscript.
